# Effects of Vagal Nerve Stimulation on Rectal Tone and Distal Colon Transit in Rats Mediated via the Vagal-Sacral Pathway

**DOI:** 10.3390/cells15111037

**Published:** 2026-06-05

**Authors:** Yan Li, Yan Wang, Shiying Li, Kaijie Wang, Jahangir Alam, Shiyuan Gong, Ying Zhu, Jiande D. Z. Chen

**Affiliations:** Division of Gastroenterology and Hepatology, Department of Internal Medicine, Medical School, University of Michigan, Ann Arbor, MI 48109, USA; yanlili@umich.edu (Y.L.); mdalam@umich.edu (J.A.);

**Keywords:** vagus nerve stimulation, distal colon, rectal tone, nitrergic signaling, cholinergic signaling, rat

## Abstract

**Highlights:**

**What are the main findings?**

- Cervical and auricular Vagal Nerve Stimulation (VNS) effectively modulates distal colonic and rectal functions in rodent models.
- VNS-mediated regulation of the lower gastrointestinal tract is driven by the coordination of cholinergic and nitrergic signaling pathways.

**What are the implications of the main findings?**

- A significant crosstalk exists between the vagal and sacral parasympathetic pathways.
- VNS is a promising therapeutic alternative for managing colorectal disorders, including Inflammatory Bowel Disease (IBD) and chronic constipation.

**Abstract:**

The vagus nerve (innervating the gut from esophagus to proximal colon) and sacral nerve (innervating distal colon and rectum) are key parasympathetic regulators of gastrointestinal (GI) function. While vagus nerve stimulation (VNS) has shown therapeutic potential in upper GI disorders, its role in modulating distal colon and rectal function remains poorly understood. This study investigated the effects and mechanisms of VNS on distal colon transit and rectal tone in rats. Adult male Sprague Dawley rats were implanted with stimulation electrodes at the cervical or auricular vagal afferent nerve. VNS was applied with varying frequencies, pulse widths, and amplitudes. Rectal tone was assessed using a barostat device, and distal colon transit was evaluated using bead expulsion. Nitrergic and cholinergic contributions were examined using L-NAME and nNOS expression, and acetylcholine ELISA and ChAT expression, respectively. Central pathways were investigated by immunofluorescence staining of c-fos and ChAT in the nucleus tractus solitarius (NTS). Sacral efferent pathway was assessed by chemogenetic inhibition of the dorsal motor nucleus of the vagus (DMV) and Barrington nucleus (BN/PMC). VNS (5 Hz, 0.1 and 0.5 ms, 0.5 mA) significantly increased rectal volume, indicating relaxation, and accelerated distal colon transit. L-NAME abolished VNS-induced rectal relaxation, while nNOS expression in the rectum was upregulated, confirming nitrergic mediation. Distal colon transit was associated with increased acetylcholine release and ChAT expression, highlighting cholinergic involvement. VNS enhanced c-fos and ChAT-positive neurons in the NTS, suggesting central integration of vagal afferent signals. Chemogenetic inhibition of DMV and BN attenuated rectal relaxation, indicating that VNS effects are mediated via a vagal–NTS–sacral pathway. VNS modulates distal colon transit and rectal tone through coordinated nitrergic and cholinergic signaling and central vagal-to-sacral circuits. These findings reveal functional crosstalk between vagal and sacral parasympathetic pathways and provide mechanistic insight into potential VNS therapy for lower GI disorders.

## 1. Introduction

The vagus and sacral nerves are two major components of the parasympathetic nervous system that coordinately regulate gastrointestinal (GI) function. Anatomically, the vagus nerve primarily innervates the upper GI tract, including the esophagus, stomach, small intestine, and proximal colon [1], whereas the sacral nerve mainly innervates the lower GI tract, particularly the distal colon, rectum, and anus, as well as other pelvic organs via the pelvic nerves [2].

The vagal input information is integrated and regulated by the dorsal vagal complex (DVC), which consists of three central nuclei: the nucleus tractus solitarius (NTS), dorsal motor nucleus of the vagus (DMV), and area postrema (AP) [3]. The NTS acts as a central hub, integrating visceral sensory signals from the gut via afferent vagal fibers [4]. The DMV contains preganglionic parasympathetic neurons that transmit signals to regulate GI contraction, motility, and secretion via efferent vagal pathways [5].

Parasympathetic regulation of the colon is mediated through either the vagal–vagal axis or the sacral–sacral axis, both centered in the NTS [6]. The proximal colon is innervated by the vagus nerve through the vagal–NTS–vagal axis [7], whereas preganglionic neurons in the lumbosacral spinal cord are primarily responsible for parasympathetic innervation of the distal colon and anorectum via the sacral nerve system [8]. Moreover, in the parasympathetic regulation, cholinergic (acetylcholine, ACh) and nitrergic (nitric oxide, NO) signaling are orchestrated to regulate GI functions in a coordinated manner [9]. Both neurotransmitters are essential mediators of parasympathetic postganglionic neuron activity, controlling GI tone and peristalsis.

Dysfunction of the vagus nerve (dysautonomia) can lead to various GI disorders, including gastroparesis, irritable bowel syndrome (IBS), and inflammatory bowel disease (IBD) [10,11]. Vagus nerve stimulation (VNS), an emerging neuromodulatory therapy, has shown promise in reducing GI inflammation and improving symptoms in IBD, including ulcerative colitis and Crohn’s disease, by modulating the vagal–vagal axis [12,13]. Mechanisms underlying VNS effects on GI disorders include modulation of the enteric nervous system to improve gastric emptying and motility [14,15], release of Ach to reduce pro-inflammatory cytokines such as TNF-α and IL-10 [16], modulation of the brain–gut axis to decrease visceral hypersensitivity in IBS [17], and indirect influence on gut microbiota composition and function, thereby altering inflammation and motility [18]. Since the vagal nerve does not innervate the distal colon and anorectum, evidence regarding possible VNS regulation of rectal tone and underlying mechanisms remains limited.

Barrington’s nucleus (BN) is a group of neurons in the brainstem that acts as the control center for the micturition reflex, also known as the pontine micturition center. It sends axons to the sacral parasympathetic nucleus in the spinal cord, where preganglionic neurons that innervate the bladder are located, making it a key link in the pathway that controls urination [19,20]. Sacral nerves, like other autonomic nerves, contain both sensory and motor components that directly innervate the distal colon, rectum, and anus, controlling their functions through connections with the pelvic splanchnic nerves and the inferior hypogastric plexus. Sacral nerve stimulation (SNS) is FDA-approved for treating fecal incontinence [21]; however, its therapeutic effects on colorectal motility have not been established [22,23]. A study in rodents has demonstrated that SNS ameliorates loperamide-induced constipation by promoting distal colon transit, mediated via autonomic-cholinergic activity [24].

Beyond their independent innervation, evidence suggests functional connections between vagal and sacral nerves, likely via interactions with central neural circuits, such as NTS and DMV [25,26]. Animal studies have shown that SNS exerts anti-inflammatory effects in TNBS-induced colitis by enhancing vagal activity through the spinal afferent–brainstem–vagal efferent–colon pathway [27]. Similarly, SNS with specific stimulation parameters improves gastric accommodation by enhancing vagal activity and promoting antral peristalsis, confirming a spinal afferent–vagal efferent pathway [28]. Prokinetic effects of SNS on stomach and small intestine function, regions not directly innervated by sacral nerves, are also mediated via sacral afferent–vagal efferent pathways [29]. On the contrary, VNS has also been shown to exert its regulatory effect on the colorectum. In patients with constipation-dominant irritable bowel syndrome (IBS-C), transcutaneous auricular VNS (taVNS) was reported to improve the overall symptoms, recto-anal inhibitory reflex, and rectal sensation to rectal distention, suggesting a vagal afferent–sacral efferent pathway [28]. In rats, VNS improved distal colon contractions via muscarinic receptors [30]. Similarly, other studies have shown that auricular VNS accelerated colon transit and enhanced enteric neural functions via the vagal afferent–central sensory nuclei–vagal efferent pathway [31,32]. Taken together, the evidence has implied a crosstalk between vagal and sacral nerves in modulating the organ function via either vagal–NTS–sacral or sacral–NTS–vagal pathways [33]. However, there is a lack of direct evidence regarding whether VNS is capable of altering the tone of the rectum that is innervated largely by the sacral nerve, and whether the perceived effect is mediated via the hindbrain.

Accordingly, the aim of this study was to test the following hypothesis: in rats, VNS is able to modulate distal colon and rectum function via a vagal–NTS–sacral pathway, and this regulation is likely mediated by the coordinated action of cholinergic and nitrergic signaling in the colorectum.

## 2. Materials and Methods

### 2.1. Animals

Eight-week-old adult male Sprague-Dawley rats were obtained from Charles River Laboratories (MA, USA). Rats were housed in the University animal facility under controlled conditions (22 °C, 65% humidity, 12 h light/dark cycle). All surgical and experimental procedures were approved by the Institutional Animal Care and Use Committee at the University of Michigan.

### 2.2. Surgical Procedures

#### 2.2.1. VNS Electrode Implantation

Following overnight fasting, 16 rats were anesthetized with 2% isoflurane mixed with oxygen (2 L/min). Hair on the neck was removed, and a midline incision was made on the left side of the neck. The vagus nerve was isolated from the carotid artery, and a pair of wire electrodes (Medtronic, Streamline TM, Minneapolis, MN, USA) was implanted circumferentially around the nerve and secured with silicone glue, with an inter-electrode distance of 3–5 mm. The electrode wires were anchored in the sternocleidomastoid muscle layer, tunneled subcutaneously, and externalized to the back of the neck. After 7–10 days of recovery, rats were used for subsequent experiments.

#### 2.2.2. aVNS Electrode Implantation

The procedure was the same as above except that two same wire electrodes were implanted unilaterally in the right ear, one at the auricular cymba concha and the other at the cavum concha.

### 2.3. Experimental Protocols

To habituate the rats to handling and experimental conditions, the rats were brought to the lab and placed in a transparent restrainer 60 min daily for a week before any experiment began. All experiments were performed while the rats were awake, and no stressful behaviors were noted.

#### 2.3.1. Experiment 1: Effects of VNS on Rectal Tone with Different Parameters

This experiment was designed to determine the effective parameters for VNS to alter rectal tone. Sixteen rats were randomized into four series of sessions with different stimulation parameters (6 rats/session): (1) VNS: 5 Hz frequency, 0.1 ms pulse width, amplitudes of 0.5, 1, 2, and 3 mA, 10 min per amplitude. (2) VNS: 5 Hz frequency, 0.3 ms pulse width, amplitudes of 0.5, 1, 2, and 3 mA, 10 min per amplitude. (3) VNS: 5 Hz frequency, 0.5 ms pulse width, amplitudes of 0.5, 1, 2, and 3 mA, 10 min per amplitude. (4) Sham-VNS: identical setup but no stimulation current. The selection of a fixed 5 Hz stimulation frequency was based on a previous study [28] in which sacral nerve stimulation at 5 Hz was more effective than 15 Hz or 30 Hz in altering the function of the rectum in rats. Rats underwent a 30 min baseline recording before stimulation. In each experimental session, a nitrile balloon (1 × 2 cm) was inserted 3 cm from the anus under brief isoflurane anesthesia; after recovery, the rat was placed in a transparent restrainer. After a 20 min adaptation period, rectal volume was continuously measured under a constant intra-rectal pressure of 10 mmHg using a computerized barostat device (Distender II, G&J Electronics Inc., Toronto, ON, Canada). Stimulation was delivered via a Universal Pulse Generator (DS8000, World Precision Instruments, Sarasota, FL, USA). Rectal volumes were analyzed to determine the most effective set of VNS parameters, which were used for subsequent experiments.

#### 2.3.2. Experiment 2: Nitrergic Mechanisms of VNS on Rectal Tone

The aim of this experiment was to determine the nitrergic mechanism involved in the inhibitory effect of VSN on rectal tone. The same 16 rats were studied in two randomized sessions (8 rats/session): (1) VNS + L-NAME: After a 20 min baseline, L-NAME (25 mg/kg, IP) was administered. Ten minutes later, VNS with the most effective parameters derived from Experiment 1 (5 Hz, 0.1 ms, 0.5 mA) was applied for 60 min. (2) Sham-VNS + L-NAME: Same procedure but with VNS of zero output current. The interval between sessions was three days.

#### 2.3.3. Experiment 3: Effects of VNS on Distal Colon Transit

This experiment was designed to study the effect of VNS on distal colon transit. Rats randomly chosen from the 16 rats in Experiment 1 underwent two randomized sessions (8 rats/session): (1) VNS: After overnight fasting, rats were acclimated in a small cage for 20 min. A 3 mm plastic bead was inserted 3 cm from the anus under brief isoflurane anesthesia. Rats were connected to the pulse generator, and after full recovery, VNS (5 Hz, 0.1 ms, 0.5 mA) was applied. Bead expulsion time was recorded. (2) Sham-VNS: Same procedure but without stimulation.

#### 2.3.4. Experiment 4: Molecular Mechanisms of VNS

At the completion of the above experiments. The 16 rats were randomly assigned to: (1) VNS (*n* = 8): VNS (5 Hz, 0.1 ms, 0.5 mA) applied for 2 h. Rectum was slightly distended with a barostat balloon (10 mmHg) during the first 30 min. (2) Sham-VNS (*n* = 8): Same procedure without stimulation. At the end of the session, the rats were euthanized, and rectum, distal colon, proximal colon, and brain (NTS) were collected for molecular analyses.

#### 2.3.5. Experiment 5: Effects of Blockade of DMV/BN by AAV Virus on Rectal Tone

The aim of this experiment was to determine the central pathway of VNS, involving the DMV and BN in six rats with AAV virus in the BN and six rats with AAV virus in both the BN and DMV.

AAV virus injection: Under isoflurane anesthesia, the rat was mounted on a stereotaxic frame. Bilateral craniotomies were performed above the DMV and/or BN. For DMV silencing, pAAV8-hSyn-DIO-hM4D(Gi)-mCherry (0.7 μL per side) was injected at 0.1 μL/min. BN silencing involved additional injections (0.5 μL per side). Needles were left in place for 10 min to prevent backflow. Craniotomies were sealed with sterile bone wax, skin sutured, and rats recovered 3 weeks before experiments.

Experimental sessions: The rats injected bilaterally with an AAV virus in the BN (*N* = 6) or in both the BN and DMV (*N* = 6) underwent two randomized sessions: a control session where they received saline, and a chemogenetic blockade session where they were administered CNO (Clozapine-N-Oxide) by IP. In each session, the rat was placed in the restrainer for 20 min acclimation after overnight fasting and received either saline or CNO. Thirty minutes later, the baseline of rectal volume was recorded for 15 min, and the rats were then exposed to auricular vagal nerve stimulation (aVNS) using the same parameters used in the VNS experiments (5 Hz, 0.1 ms, 0.5 mA) for 30 min. Rectal volume was continuously recorded during the stimulation.

### 2.4. Measurements

#### 2.4.1. Rectal Tone

Rectal tone was measured as changes in rectal volume under a constant pressure (10 mmHg) using a computerized barostat. Colorectum was cleansed using a 1 mL glycerol enema 30 min before balloon insertion (1 × 2 cm nitrile balloon, 3 cm from anus). Rats were placed in a transparent restrainer during measurement. An increase in rectal volume indicated decreased tone [34].

#### 2.4.2. Distal Colon Transit Time (dCTT)

dCTT was assessed using bead expulsion time [24,35]. A single 3 mm bead was inserted 3 cm from the anus. Time from insertion to expulsion was recorded as dCTT.

#### 2.4.3. Tissue Collection

Colon and rectum tissues for Western blot and ELISA were stored at −80 °C; tissues for histology and immunofluorescence were fixed in 4% paraformaldehyde for 6 h and stored in 70% ethanol. Brain tissue was perfused with 1× PBS, fixed with 4% paraformaldehyde for 24 h, and cryoprotected in 10%, 20%, and 30% sucrose solutions (24 h each) for frozen sections.

#### 2.4.4. Acetylcholine ELISA

Colon tissues were homogenized in 1× PBS using an electrical homogenizer, incubated at 4 °C for 30 min, and centrifuged. Supernatants were analyzed with the Ach ELISA kit (NBP2-66389, NOVUS Biologicals, Centennial, CO, USA) following the manufacturer’s instructions.

### 2.5. Western Blotting

Colon tissues stored at −80 °C were homogenized in RIPA buffer supplemented with a protease inhibitor cocktail using an electrical homogenizer (Lab GEN 125, Cole-Parmer, Vernon Hills, IL, USA). Following centrifugation, the supernatants were collected and protein concentrations were determined. Samples were balanced, and 50 µg of total protein per sample were loaded onto a 4–20% SDS-PAGE gel for protein separation [20].

The separated proteins were transferred to a PVDF membrane (Bio-Rad, South Granville, NSW, Australia) and blocked with 5% non-fat dry milk. Membranes were then incubated overnight with the following primary antibodies: neural nitric oxide synthase (nNOS) (1:500, #4231S, Cell Signaling, Danvers, MA, USA), choline acetyltransferase (ChAT) (1:1000, #ab181023, Abcam, Waltham, MA, USA), and GAPDH (1:3000, #5174S, Cell Signaling). After incubation with the appropriate secondary antibody, membranes were imaged using a ChemiDoc Digital Image System 1000 (Bio-Rad). Protein band density was quantified using ImageJ2 software (NIH).

### 2.6. Immunofluorescence Staining

#### 2.6.1. Rectal Tissue

For ChAT immunostaining in paraffin-embedded rectal sections [31], slides were rehydrated through a graded series of xylene and ethanol (100%, 95%, 75%, 50%), followed by 1× PBS. After blocking with 5% BSA, sections were incubated in primary ChAT antibody (1:500, #ab181023, Abcam) in a humidified chamber at 4 °C overnight. Sections were subsequently incubated with Alexa Fluor-594 secondary antibody (A21207, Invitrogen, Waltham, MA, USA) and counterstained with DAPI (Invitrogen). Imaging was performed using a fluorescence microscope (Nikon, Rhodes, NSW, Australia, Eclipse TE2000-S).

#### 2.6.2. Brain Tissue

Frozen brain tissues were coronally sectioned using a cryostat (Leica CM1900, Wetzlar, Germany) according to bregma coordinates (NTS: −15 to −13 mm) [36]. Sections were treated with 30% H_2_O_2_ solution to quench autofluorescence and permeabilized with 0.5% Triton X-100 for 15 min. After blocking with 5% BSA, sections were incubated overnight at 4 °C with primary antibodies against C-fos (1:300, ab208942, Abcam) and ChAT (1:500, #ab181023, Abcam). Following incubation with secondary antibodies (Alexa Fluor-488 #A21202 and Alexa Fluor-594, Invitrogen), images were captured using a fluorescence microscope. The number of positively stained cells was quantified using ImageJ2 (NIH), with three images analyzed per rat (*n* = 6 rats per group).

### 2.7. Statistical Analysis

Data are presented as mean ± SD. Statistical significance was assessed using one-way ANOVA with Tukey’s post hoc test or Student’s *t*-test. *p* ≤ 0.05 was considered significant.

### 2.8. Data Availability

The datasets generated and analyzed during the current study are included in this manuscript and are available from the corresponding author upon request.

## 3. Results

### 3.1. VNS Induced Rectal Relaxation and Nitrergic Mechanisms

**Figure 1:**
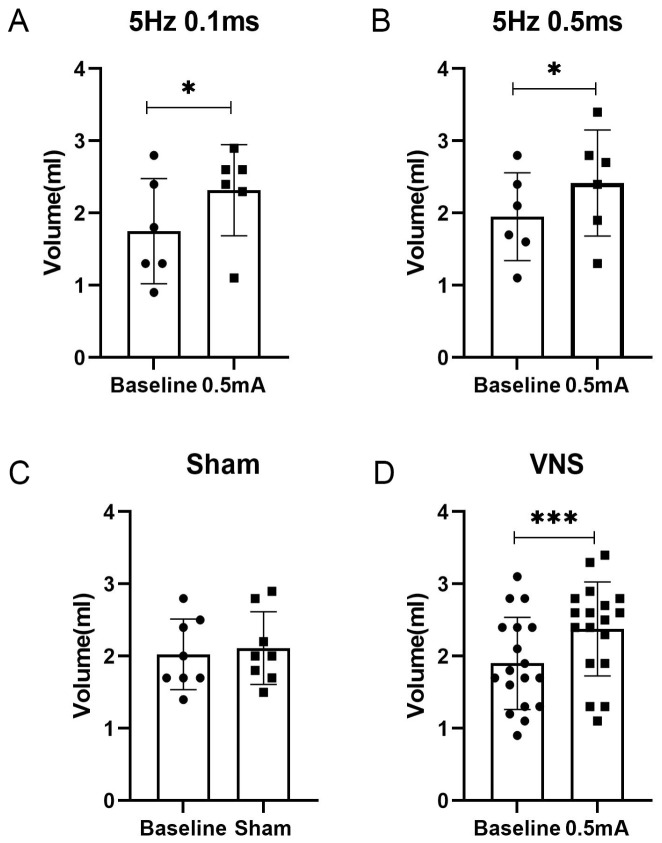
Effect of cervical VNS on rectal tone in normal rats. (**A**) VNS with 5 Hz, 0.1 ms and 0.5 mA for 10 min (Baseline vs. VNS, *p* = 0.037, *N* = 6); (**B**) VNS with 5 Hz, 0.5 ms and 0.5 mA for 10 min (Baseline vs. VNS, *p* = 0.038, *N* = 6); (**C**) sham-VNS with no VNS and 0 mA current output (Baseline vs. VNS, *p* = 0.6, *N* = 8); (**D**) data from all the VNS pulse widths of 0.1, 0.3, 0.5 ms and 0.5 mA (Baseline vs. VNS, *p* = 0.0008, *N* = 18). * Represents significance at *p* ≤ 0.05; *** represents significance at *p* ≤ 0.001.

VNS significantly increased rectal volume under specific parameters. As shown in Figure 1A, VNS at 5 Hz, 0.1 ms, and 0.5 mA increased rectal volume compared to baseline (*p* = 0.037), as did VNS at 5 Hz, 0.5 ms, and 0.5 mA (*p* = 0.038, Figure 1B). No significant change was observed with sham-VNS (*p* = 0.60, Figure 1C).

When data from all three VNS pulse widths (0.1, 0.3, and 0.5 ms) were combined, a more pronounced increase in rectal volume was observed compared to the baseline (*p* < 0.0008, Figure 1D). However, no significant differences were detected between VNS and sham-VNS at amplitudes of 1, 2, and 3 mA.

**Figure 2:**
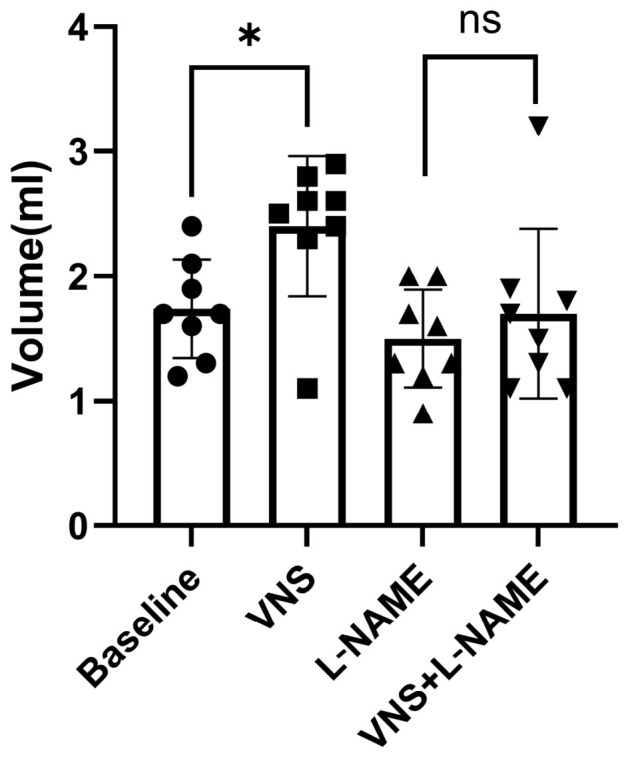
Effect of L-NAME on the rectal tone responding to the cervical VNS. The rectal volume was altered by VNS and L-NAME treatment labeled in the baseline, VNS (0.1 ms), L-NAME (Baseline + L-NAME) and VNS + L-NAME. Comparisons between the Baseline and VNS (*p* = 0.016, *N* = 8), and between L-NAME and VNS + L-NAME (*p* = 0.48, *N* = 8), are shown. * Represents significance at *p* ≤ 0.05; “ns” represents no significance.

Nitric oxide (NO) is known to mediate rectal relaxation. To examine the role of NO in VNS-induced effects, the NO synthase inhibitor L-NAME was administered. As shown in Figure 2, L-NAME completely blocked VNS-induced rectal relaxation. No significant difference was observed between L-NAME alone and L-NAME plus VNS, indicating that VNS-induced relaxation is NO-dependent.

**Figure 3:**
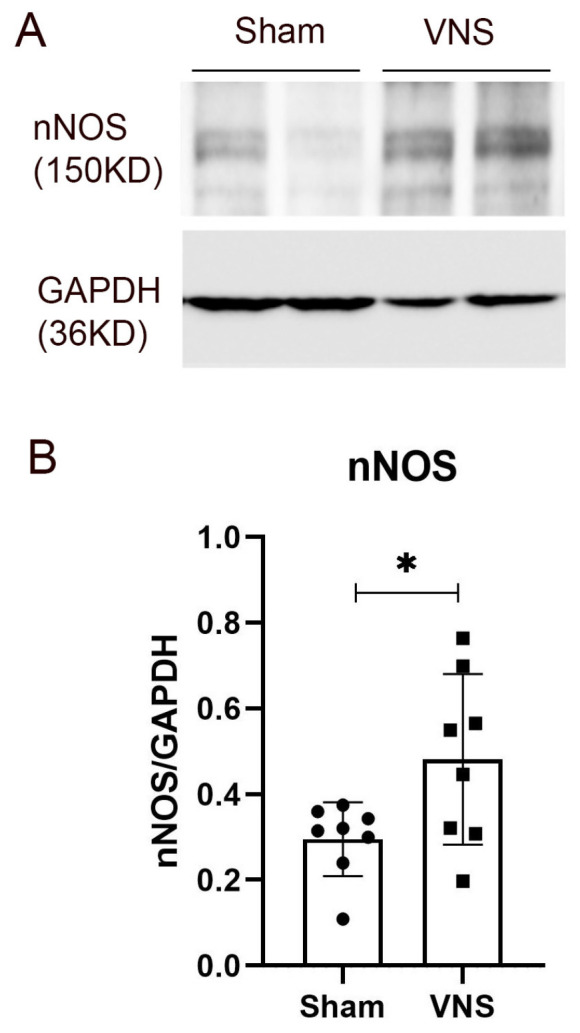
Activation of nNOS in the rectum in response to cervical VNS. (**A**) Expression of nNOS measured by Western blot (WB). (**B**) The quantification of nNOS band density normalized by GAPDH from the WB in A (Sham vs. VNS, *p* = 0.029, *N* = 8). * Indicates significant difference in statistical analysis (*p* ≤ 0.05).

Western blot analysis revealed that the nNOS expression in the VNS group was higher than in the sham group (*p* ≤ 0.029, Figure 3A,B).

### 3.2. VNS Induced Acceleration of Distal Colon Transit and Cholinergic Mechanisms

**Figure 4:**
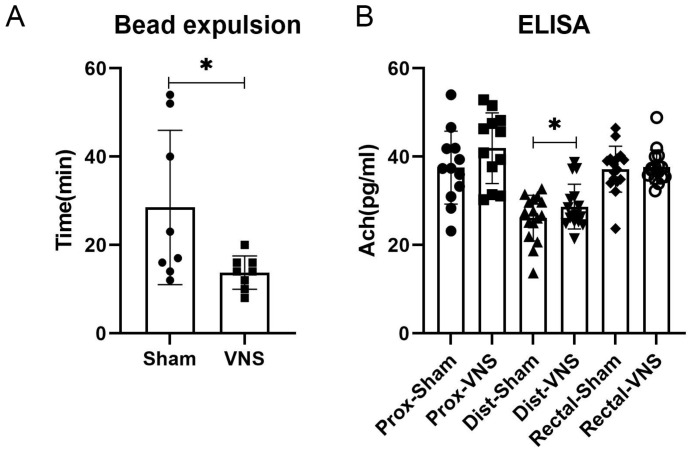
Response of distal colon transit to the cervical VNS. (**A**) Measurement of bead expulsion time (Sham vs. VNS, *p* = 0.03, *N* = 8). (**B**) The ELISA assay of acetylcholine (AcH) in the different colon tissues (Sham vs. VNS in proximal colon, *p* = 0.24; Sham vs. VNS in distal colon, *p* = 0.027; Sham vs. VNS in rectum, *p* = 0.69, *N* = 6). * Represents the significant difference in statistical analysis (*p* ≤ 0.05).

VNS significantly reduced bead expulsion time compared to sham-VNS (*p* < 0.05, Figure 4A), indicating improved distal colon motility. ELISA analysis of ACh showed a significant increase in distal colon tissue after VNS compared to sham-VNS (*p* = 0.027, Figure 4B), whereas no significant changes were observed in the rectum or proximal colon.

**Figure 5:**
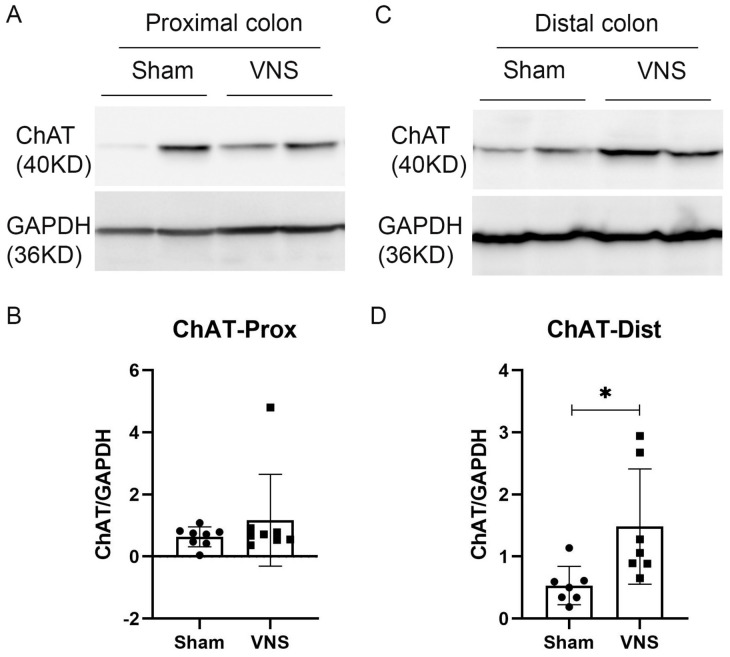
Expression of ChAT in proximal and distal colons induced by cervical VNS. (**A**) ChAT expression in the proximal colon assessed by Western blot (WB). (**B**) The quantification of ChAT band density normalized by GAPDH from the WB in the proximal colon (Sham vs. VNS, *p* = 0.38, *N* = 8). (**C**) ChAT expression in the distal colon assessed by Western blot (WB). (**D**) The quantification of ChAT band density normalized by GAPDH from the WB in the distal colon (Sham vs. VNS, *p* = 0.024, *N* = 8). * Indicates significant difference in statistical analysis (*p* ≤ 0.05).

Western blot analysis revealed that ChAT expression in the distal colon was significantly increased by VNS compared to sham treatment (Figure 5C,D), whereas no difference was observed in the proximal colon (Figure 5A,B). These findings suggest that VNS enhances distal colon transit via cholinergic activation and increased ACh synthesis.

### 3.3. Central and Sacral Pathways of VNS

**Figure 6:**
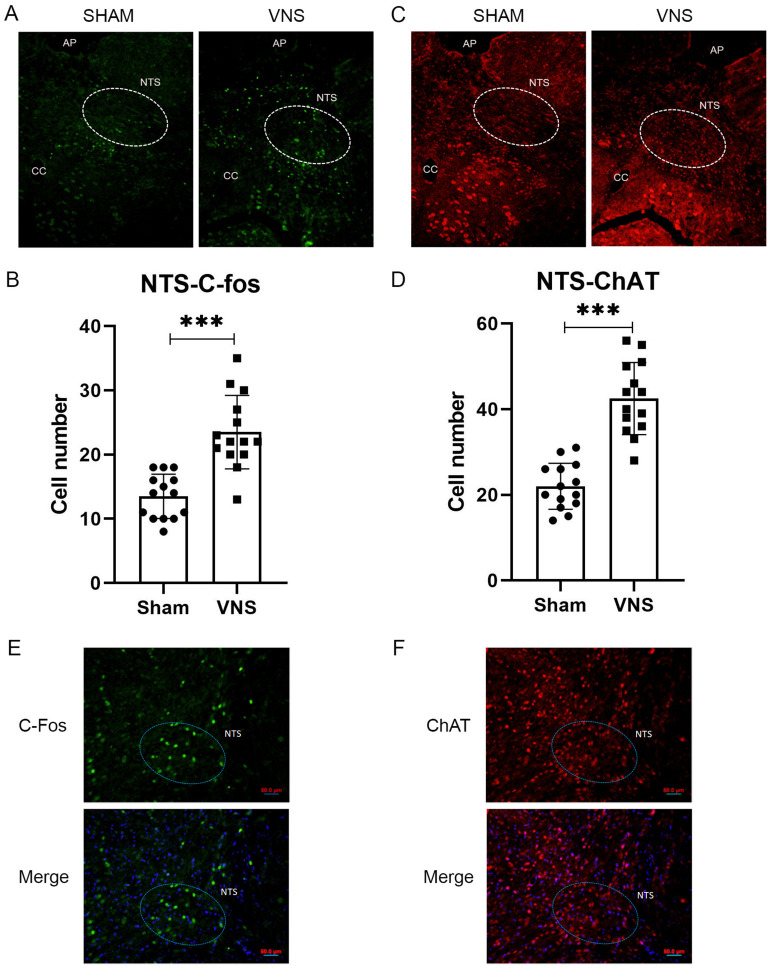
Activation of C-fos and ChAT in brain NTS in response to cervical VNS. (**A**) Immunofluorescene staining of C-fos (Green) using the C-fos antibody in NTS. (**B**) The quantification of C-fos positive-stained cells in the NTS (Sham vs. VNS, *p* ≤ 0.0001, *N* = 8). (**C**) Immunofluorescene staining of ChAT (Red) using ChAT antibody in the NTS. (**D**) The quantification of ChAT positive-stained cells in the NTS (Sham vs. VNS, *p* ≤ 0.0001, *N* = 8). *** Represents significant difference in statistical analysis (*p* ≤ 0.0001). Scale bar: 50 µm. (**E**) Representative high-magnification immunofluorescence images of the central NTS from the VNS group, stained for C-Fos. (**F**) Representative high-magnification immunofluorescence images of the central NTS from the VNS group, stained for ChAT.

The NTS is known to integrate visceral sensory signals. To identify central mechanisms, C-fos (neuronal activity marker) and ChAT expression were analyzed in the NTS by immunofluorescence. VNS significantly increased C-fos-positive cells compared to sham-VNS (Figure 6A,B). Similarly, ChAT-positive neurons in the NTS were more abundant in VNS-treated rats than in controls (Figure 6C,D).

**Figure 7:**
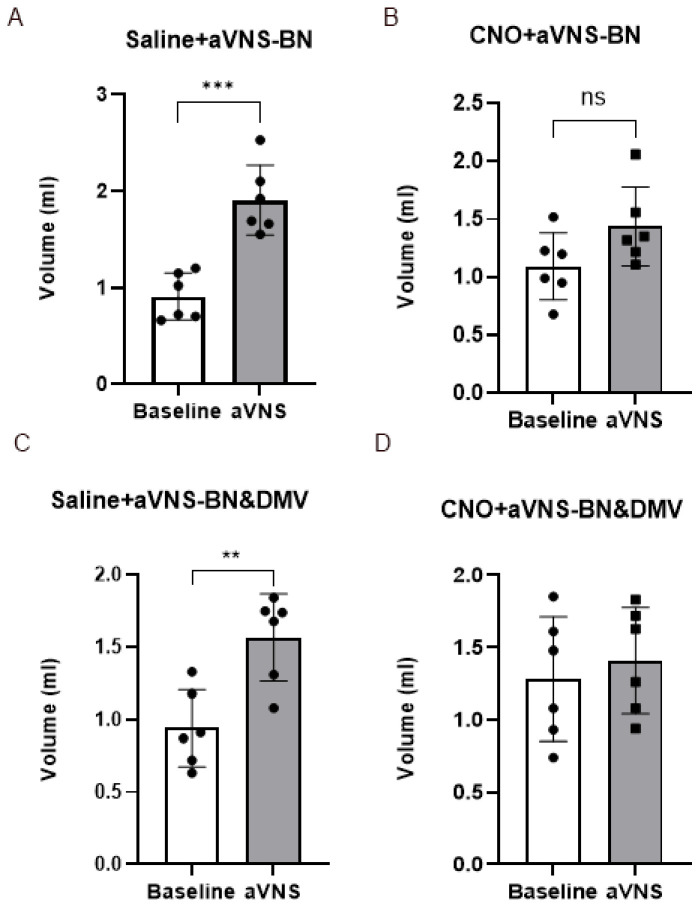
Roles of the BN and DMV in regulating rectal tone. (**A**) Effect of aVNS on rectal tone following saline administration (Control). aVNS significantly elevated the rectal volume (*p* = 0.0002, *N* = 6). (**B**) Partial inhibition of aVNS-induced rectal volume increases following chemogenetic blockade of the BN (CNO administered) (*p* = 0.089, *N* = 6). (**C**) Significant increase in rectal volume induced by aVNS in the control (saline-administered) group where both the BN and DMV were targeted for DREADD expression (*p* = 0.0035, *N* = 6). (**D**) Complete abolition of the aVNS-induced rectal volume increase (rectal relaxation) following chemogenetic blockade of both the BN and DMV (CNO administered) (*p* = 0.59, *N* = 6). ** Represents significance at *p* ≤ 0.01; *** represents significance at *p* ≤ 0.001; “ns” indicates no statistical significance.

To further explore the VNS–NTS-dependent sacral pathway, chemogenetic inhibition was performed on the BN alone and both the BN and DMV using hM4D DREADDs. In rats chronically injected with AAV virus in the BN alone, aVNS substantially increased rectal volume compared to baseline in the saline session (Figure 7A); this increase was substantially blocked in the CNO session (Figure 7B). In rats chronically injected with AAV virus in both the BN and DMV, aVNS solicited a similar significant increase in rectal volume in the saline session (Figure 7C); this increase was completely blocked when the rats were treated with CNO (Figure 7D).

## 4. Discussion

In this study, we used rectal barostat, a standard technique for assessing rectal sensory and motor function, to evaluate the rectal response to VNS [37,38]. We found that cervical VNS with parameters of 5 Hz, 0.5 mA, and 0.1 ms was most effective in inhibiting rectal tone in normal rats. Using this set of parameters, VNS substantially and significantly inhibited rectal tone, mediated via the nitrergic mechanism and accelerated distal colon transit via the cholinergic mechanisms. Similarly, aVNS using the same parameters was also able to significantly and substantially inhibit rectal tone, and the effect was substantially blocked by chemogenetic blocking of the BN and completely blocked by chemogenetic blocking of both the BN and DMV, suggesting a vagal–sacral pathway.

The effects of VNS depend on stimulation parameters. Low-frequency VNS (1–10 Hz) has been reported to activate anti-inflammatory pathways by the release of Ach [39,40] and improve GI motility [41]. In contrast, a few studies have suggested that high-frequency stimulation (100 Hz) may be more effective in reducing visceral hypersensitivity in rodent models [42,43]. However, there is no consensus on standardized VNS parameters for universal or specific targets. To optimize the effect of VNS on rectal tone in normal rats, we conducted four series of experimental sessions using low frequency (5 Hz) with varying pulse widths (0.1, 0.3, 0.5 ms) and amplitudes (0.5, 1, 2, 3 mA), respectively. We found that both the parameter sets of 5 Hz, 0.1 ms, and 0.5 mA, and 5 Hz, 0.5 ms, and 0.5 mA significantly increased rectal volume, indicating an inhibitory role of VNS on rectal tone. Consequently, the parameters of 5 Hz, 0.1 ms, and 0.5 mA were selected for further acute VNS experiments in this study.

In addition to rectal function, we investigated whether VNS also affected the distal colon function, as the sacral nerve endogenously innervates the lower GI tract, including the colorectum. The dCTT revealed that acute VNS significantly decreased bead expulsion time compared to sham stimulation, suggesting enhanced distal colon motility. These results aligned with previous studies in which VNS was reported to improve colonic motility in rats with opioid-induced constipation [31] and alleviate colon inflammation in rats with TNBS-induced colitis [44]. Preclinical and clinical trials have demonstrated the therapeutic effects of taVNS on abdominal pain, IBS, and IBD [28,45,46,47]. For example, in a recent study in patients with IBS-C, taVNS was reported to improve rectal sensation in addition to alleviating overall IBS symptoms by enhancing parasympathetic activity [28]. Taken together, electrical stimulation at both the cervical vagal nerve and auricular vagal nerve seems to effectively modulate both distal colon and rectum function.

Activation of the vagal nerve is known to result in the release of ACh in the target organ. Ach, a neurotransmitter, is a primary promoter of GI motility; it also inhibits GI inflammation by suppressing pro-inflammatory cytokine production and immune responses [48]. To determine whether ACh mediates VNS effects in the distal colon and rectum, we measured ACh levels in the proximal colon, distal colon, and rectum tissues by ELISA. Compared to sham stimulation, VNS significantly increased Ach in the distal colon but not in the rectum or proximal colon, consistent with the observed physiological response, implying thar a cholinergic signaling occurred in the distal colon. Previous studies have suggested that VNS-induced Ach release in the distal colon promotes anti-inflammatory effects by inhibiting pro-inflammatory cytokines (TNF-α, IL-6) and suppressing immune activation via α7 nicotinic Ach receptors (α7nAChR) on macrophages and other immune cells [49,50]. Interestingly, our data indicated that ACh levels in the proximal colon did not significantly respond to VNS. This was unexpected, as cervical VNS has been shown to evoke contractions throughout the colon via the release of ACh [30]. On the other hand, however, this was conceivable since the experimental manipulation was performed in the rectum (30 min rectal distention) and not in the proximal colon, meaning that the condition of the proximal colon was normal during VNS. As expected, VNS increased ChAT expression in the distal colon, confirming further cholinergic signaling involvement in VNS-dependent colonic responses. ChAT activation in the enteric nervous system (ENS) is associated with contraction, supporting a cholinergic mechanism for VNS-induced modulation of distal colon and rectum function [51,52].

In addition to ACh, nitric oxide is a key neuromodulator in the GI tract, regulating smooth muscle relaxation, mucosal defense, blood flow, and motility. Vagal nerve activation has been reported to trigger NO production in the GI system [53,54]. To test the role of NO in VNS-induced rectal relaxation, we used the NO synthase inhibitor L-NAME. L-NAME treatment blocked VNS-induced rectal relaxation, indicating that nitrergic signaling contributes to VNS-mediated modulation. Furthermore, VNS increased expression and activation of nNOS, the NO-producing enzyme, in the rectum, confirming that VNS-induced rectal modulation involves NO release and intergenic signaling.

The NTS and DMV are key brainstem nuclei integrating visceral sensory information to regulate autonomic functions [55]. Activation of the NTS reflects afferent vagal signaling, and c-fos expression is a marker of neuronal activity [25]. High c-fos expression in NTS neurons following VNS indicates robust vagal activation. Interestingly, VNS also increased ChAT expression in the NTS, suggesting activation of cholinergic neurons. Although direct evidence linking vagal activation to ChAT neurons in the NTS is lacking, recently, one study has reported that allergen-induced activation of Trpv1^+^ vagal sensory neurons can activate Dbh^+^ neurons in the NTS, which subsequently project to the nucleus ambiguus, activating ChAT neurons, indicating a potential indirect vagal–NTS–ChAT pathway [56].

Barrington’s nucleus is a brain region known to regulate colorectal function [19,57]. It projects to both the sacral parasympathetic nucleus in the spinal cord, which innervates the descending colon, and to the noradrenergic locus coeruleus [58,59]. Activation of Barrington’s nucleus stimulates colonic transit and motility via the autonomic nervous system [58]. Conversely, inhibiting Barrington’s nucleus can disrupt the normal micturition reflex, leading to urinary retention and altered bowel motility [19,60]. Stimulation techniques, such as micro stimulation or optogenetic activation, was reported to trigger bladder contraction and micturition, highlighting its excitatory role in this reflex [19]. In our study, both VNS and aVNS were shown to play a substantial role in modulating the colorectum function. The chemogenetic silencing of BN neurons substantially blocked the rectal tone response induced by aVNS, demonstrating the involvement of the BN–sacral efferent pathway. Furthermore, silencing both BN and DMV neurons led to a complete block of aVNS-induced rectal relaxation, suggesting the involvement of both sacral efferent (major) and vagal efferent (minor) pathways.

Parasympathetic pathways, including vagal–NTS–vagal and sacral–NTS–sacral, are major circuits modulating GI function. The vagal–NTS–vagal pathway primarily regulates the stomach, proximal small intestine, and proximal colon [61], while the sacral–NTS–sacral pathway endogenously controls the lower GI, such as the distal colon and rectum [62]. Recent studies have revealed the interaction between vagal and sacral nerves in the dorsal vagal complex (DVC), likely forming either vagal afferent–brainstem–sacral efferent or sacral afferent–brainstem–vagal efferent pathways [33]. In this study, our data clearly demonstrated that VNS regulates colorectum function via a vagal–NTS–sacral axis, coordinated by cholinergic and nitrergic signaling, highlighting that vagal afferent signals were transduced to the NTS, where they were projected to the DMV and BN. These nuclei then translated the signals into cholinergic and nitrergic activity within the ENS, which modulated distal colon and rectal function largely via the sacral efferent nerves.

A limitation of the current study is the focus on acute VNS effects. While our results demonstrate a clear immediate relationship between VNS and the distal colon and rectum, the physiological responses of the lower GI tract to VNS can evolve significantly over time due to neural plasticity or habituation. Chronic stimulation studies are required to determine if the observed regulatory effects are sustained or if compensatory mechanisms—such as sympathetic counter-regulation—alter the long-term therapeutic window. Future studies may explore the therapeutic potential of VNS, especially aVNS, on conditions related to reduced rectal accommodation, such as diarrhea attributed to reduced rectal compliance.

In conclusion, VNS modulates distal colon transit and rectal tone through coordinated nitrergic and cholinergic signaling and central vagal–to–sacral circuits. These findings reveal functional crosstalk between vagal and sacral parasympathetic pathways and provide mechanistic insight into potential VNS therapy for lower GI disorders.

## Data Availability

The original contributions presented in this study are included in the article. Further inquiries can be directed to the corresponding authors.

## Abbreviations

The following abbreviations are used in this manuscript:

VNS	vagal nerve stimulation
NTS	nucleus tractus solitarius
DMV	dorsal motor nucleus of the vagus
BN	Barrington’s nucleus
ENS	enteric nervous system
ChAT	choline acetyltransferase (cholinergic marker)
nNOS	neuronal nitric oxide synthase (nitrergic marker)
L-NAME	NO synthase inhibitor

## References

[1] Altschuler S.M., Escardo J., Lynn R.B., Miselis R.R. (1993). The central organization of the vagus nerve innervating the colon of the rat. Gastroenterology.

[2] Wehrwein E.A., Orer H.S., Barman S.M. (2016). Overview of the Anatomy, Physiology, and Pharmacology of the Autonomic Nervous System. Compr. Physiol..

[3] Powley T.L. (2021). Brain-gut communication: Vagovagal reflexes interconnect the two “brains”. Am. J. Physiol. Gastrointest. Liver Physiol..

[4] Chan R.K., Jarvina E.V., Sawchenko P.E. (2000). Effects of selective sinoaortic denervations on phenylephrine-induced activational responses in the nucleus of the solitary tract. Neuroscience.

[5] Browning K.N., Carson K.E. (2021). Central Neurocircuits Regulating Food Intake in Response to Gut Inputs-Preclinical Evidence. Nutrients.

[6] Browning K.N., Travagli R.A. (2014). Central nervous system control of gastrointestinal motility and secretion and modulation of gastrointestinal functions. Compr. Physiol..

[7] Yu C.D., Xu Q.J., Chang R.B. (2020). Vagal sensory neurons and gut-brain signaling. Curr. Opin. Neurobiol..

[8] McCorry L.K. (2007). Physiology of the autonomic nervous system. Am. J. Pharm. Educ..

[9] Hedlund P., Alm P., Andersson K.E. (1999). NO synthase in cholinergic nerves and NO-induced relaxation in the rat isolated corpus cavernosum. Br. J. Pharmacol..

[10] Yin J. (2025). Transcutaneous Vagal Nerve Stimulation for Gastrointestinal Disorders. J. Transl. Gastroenterol..

[11] Pellissier S., Dantzer C., Mondillon L., Trocme C., Gauchez A.S., Ducros V., Mathieu N., Toussaint B., Fournier A., Canini F. (2014). Relationship between vagal tone, cortisol, TNF-alpha, epinephrine and negative affects in Crohn’s disease and irritable bowel syndrome. PLoS ONE.

[12] Veldman F., Hawinkels K., Keszthelyi D. (2025). Efficacy of vagus nerve stimulation in gastrointestinal disorders: A systematic review. Gastroenterol. Rep..

[13] Breit S., Kupferberg A., Rogler G., Hasler G. (2018). Vagus Nerve as Modulator of the Brain-Gut Axis in Psychiatric and Inflammatory Disorders. Front. Psychiatry.

[14] Bonaz B., Sinniger V., Pellissier S. (2021). Therapeutic Potential of Vagus Nerve Stimulation for Inflammatory Bowel Diseases. Front. Neurosci..

[15] Bonaz B. (2022). Anti-inflammatory effects of vagal nerve stimulation with a special attention to intestinal barrier dysfunction. Neurogastroenterol. Motil..

[16] Berthoud H.R., Neuhuber W.L. (2019). Vagal mechanisms as neuromodulatory targets for the treatment of metabolic disease. Ann. N. Y. Acad. Sci..

[17] Yan Q., Chen J., Ren X., Song Y., Xu J., Xuan S., Jiang X., Kuang Z., Tang Z. (2023). Vagus Nerve Stimulation Relives Irritable Bowel Syndrome and the Associated Depression via alpha7nAChR-mediated Anti-inflammatory Pathway. Neuroscience.

[18] Morais L.H., Schreiber H.L.t., Mazmanian S.K. (2021). The gut microbiota-brain axis in behaviour and brain disorders. Nat. Rev. Microbiol..

[19] Pavcovich L.A., Yang M., Miselis R.R., Valentino R.J. (1998). Novel role for the pontine micturition center, Barrington’s nucleus: Evidence for coordination of colonic and forebrain activity. Brain Res..

[20] Sasaki M. (2005). Role of Barrington’s nucleus in micturition. J. Comp. Neurol..

[21] Thaha M.A., Abukar A.A., Thin N.N., Ramsanahie A., Knowles C.H. (2015). Sacral nerve stimulation for faecal incontinence and constipation in adults. Cochrane Database Syst. Rev..

[22] Carrington E.V., Evers J., Grossi U., Dinning P.G., Scott S.M., O’Connell P.R., Jones J.F., Knowles C.H. (2014). A systematic review of sacral nerve stimulation mechanisms in the treatment of fecal incontinence and constipation. Neurogastroenterol. Motil..

[23] Wang X., Chen J.D. (2023). Therapeutic potential and mechanisms of sacral nerve stimulation for gastrointestinal diseases. J. Transl. Int. Med..

[24] Huang Z., Li S., Foreman R.D., Yin J., Dai N., Chen J.D.Z. (2019). Sacral nerve stimulation with appropriate parameters improves constipation in rats by enhancing colon motility mediated via the autonomic-cholinergic mechanisms. Am. J. Physiol. Gastrointest. Liver Physiol..

[25] Bonaz B., Sinniger V., Pellissier S. (2019). Vagus Nerve Stimulation at the Interface of Brain-Gut Interactions. Cold Spring Harb. Perspect. Med..

[26] Pasricha T.S., Zhang H., Zhang N., Chen J.D.Z. (2020). Sacral nerve stimulation prompts vagally-mediated amelioration of rodent colitis. Physiol. Rep..

[27] Tu L., Gharibani P., Zhang N., Yin J., Chen J.D. (2020). Anti-inflammatory effects of sacral nerve stimulation: A novel spinal afferent and vagal efferent pathway. Am. J. Physiol. Gastrointest. Liver Physiol..

[28] Shi X., Hu Y., Zhang B., Li W., Chen J.D., Liu F. (2021). Ameliorating effects and mechanisms of transcutaneous auricular vagal nerve stimulation on abdominal pain and constipation. JCI Insight.

[29] Wang X., Zhang S., Pasricha P.J., Chen J.D.Z. (2020). Ameliorating effects of sacral neuromodulation on gastric and small intestinal dysmotility mediated via a sacral afferent-vagal efferent pathway. Neurogastroenterol. Motil..

[30] Tong W.D., Ridolfi T.J., Kosinski L., Ludwig K., Takahashi T. (2010). Effects of autonomic nerve stimulation on colorectal motility in rats. Neurogastroenterol. Motil..

[31] Zhang Y., Lu T., Meng Y., Maisiyiti A., Dong Y., Li S., Chen Y., Yin J., Chen J.D.Z. (2021). Auricular Vagal Nerve Stimulation Improves Constipation by Enhancing Colon Motility via the Central-Vagal Efferent Pathway in Opioid-Induced Constipated Rats. Neuromodulation.

[32] Hou L., Rong P., Yang Y., Fang J., Wang J., Wang Y., Zhang J., Zhang S., Zhang Z., Chen J.D.Z. (2023). Auricular Vagus Nerve Stimulation Improves Visceral Hypersensitivity and Gastric Motility and Depression-like Behaviors via Vago-Vagal Pathway in a Rat Model of Functional Dyspepsia. Brain Sci..

[33] Chen J.D. (2023). Parasympathetic control of gastrointestinal motility and cross-branch actions of parasympathetic neuromodulation. Chin. Med. J..

[34] Chen Y., Guo Y., Gharibani P., Chen J., Selaru F.M., Chen J.D.Z. (2021). Transitional changes in gastrointestinal transit and rectal sensitivity from active to recovery of inflammation in a rodent model of colitis. Sci. Rep..

[35] Million M., Maillot C., Saunders P., Rivier J., Vale W., Tache Y. (2002). Human urocortin II, a new CRF-related peptide, displays selective CRF(2)-mediated action on gastric transit in rats. Am. J. Physiol. Gastrointest. Liver Physiol..

[36] Li S., Lei Y., Chen J.D.Z. (2018). Chemotherapy-Induced Pica in Rats Reduced by Electroacupuncture. Neuromodulation.

[37] Carrington E.V., Scott S.M., Bharucha A., Mion F., Remes-Troche J.M., Malcolm A., Heinrich H., Fox M., Rao S.S., the International Anorectal Physiology Working Group and the International Working Group for Disorders of Gastrointestinal Motility and Function (2018). Expert consensus document: Advances in the evaluation of anorectal function. Nat. Rev. Gastroenterol. Hepatol..

[38] Marinica Grando L., Halfvarson J., van Nieuwenhoven M. (2024). Rectal Sensory and Compliance Testing: A Method Comparison Study Between High-Resolution Anorectal Manometry and Barostat Investigations. Diagnostics.

[39] Borovikova L.V., Ivanova S., Zhang M., Yang H., Botchkina G.I., Watkins L.R., Wang H., Abumrad N., Eaton J.W., Tracey K.J. (2000). Vagus nerve stimulation attenuates the systemic inflammatory response to endotoxin. Nature.

[40] Meregnani J., Clarencon D., Vivier M., Peinnequin A., Mouret C., Sinniger V., Picq C., Job A., Canini F., Jacquier-Sarlin M. (2011). Anti-inflammatory effect of vagus nerve stimulation in a rat model of inflammatory bowel disease. Auton. Neurosci..

[41] Lu K.H., Cao J., Oleson S., Ward M.P., Phillips R.J., Powley T.L., Liu Z. (2018). Vagus nerve stimulation promotes gastric emptying by increasing pyloric opening measured with magnetic resonance imaging. Neurogastroenterol. Motil..

[42] Zhou J., Li S., Wang Y., Lei Y., Foreman R.D., Yin J., Chen J.D. (2017). Effects and mechanisms of auricular electroacupuncture on gastric hypersensitivity in a rodent model of functional dyspepsia. PLoS ONE.

[43] Hou L.W., Fang J.L., Zhang J.L., Wang L., Wu D., Wang J.Y., Wu M.Z., Rong P.J. (2022). Auricular Vagus Nerve Stimulation Ameliorates Functional Dyspepsia with Depressive-like Behavior and Inhibits the Hypothalamus-Pituitary-Adrenal Axis in a Rat Model. Dig. Dis. Sci..

[44] Jin H., Guo J., Liu J., Lyu B., Foreman R.D., Yin J., Shi Z., Chen J.D.Z. (2017). Anti-inflammatory effects and mechanisms of vagal nerve stimulation combined with electroacupuncture in a rodent model of TNBS-induced colitis. Am. J. Physiol. Gastrointest. Liver Physiol..

[45] Krasaelap A., Sood M.R., Li B.U.K., Unteutsch R., Yan K., Nugent M., Simpson P., Kovacic K. (2020). Efficacy of Auricular Neurostimulation in Adolescents with Irritable Bowel Syndrome in a Randomized, Double-Blind Trial. Clin. Gastroenterol. Hepatol..

[46] Colombel J.F., Shin A., Gibson P.R. (2019). AGA Clinical Practice Update on Functional Gastrointestinal Symptoms in Patients with Inflammatory Bowel Disease: Expert Review. Clin. Gastroenterol. Hepatol..

[47] Shi X., Zhao L., Luo H., Deng H., Wang X., Ren G., Zhang L., Tao Q., Liang S., Liu N. (2024). Transcutaneous Auricular Vagal Nerve Stimulation Is Effective for the Treatment of Functional Dyspepsia: A Multicenter, Randomized Controlled Study. Am. J. Gastroenterol..

[48] Zheng W., Song H., Luo Z., Wu H., Chen L., Wang Y., Cui H., Zhang Y., Wang B., Li W. (2021). Acetylcholine ameliorates colitis by promoting IL-10 secretion of monocytic myeloid-derived suppressor cells through the nAChR/ERK pathway. Proc. Natl. Acad. Sci. USA.

[49] Cailotto C., Gomez-Pinilla P.J., Costes L.M., van der Vliet J., Di Giovangiulio M., Nemethova A., Matteoli G., Boeckxstaens G.E. (2014). Neuro-anatomical evidence indicating indirect modulation of macrophages by vagal efferents in the intestine but not in the spleen. PLoS ONE.

[50] Rosas-Ballina M., Ochani M., Parrish W.R., Ochani K., Harris Y.T., Huston J.M., Chavan S., Tracey K.J. (2008). Splenic nerve is required for cholinergic antiinflammatory pathway control of TNF in endotoxemia. Proc. Natl. Acad. Sci. USA.

[51] Wood J.D. (2016). Enteric Nervous System: Neuropathic Gastrointestinal Motility. Dig. Dis. Sci..

[52] Johnson C.D., Barlow-Anacker A.J., Pierre J.F., Touw K., Erickson C.S., Furness J.B., Epstein M.L., Gosain A. (2018). Deletion of choline acetyltransferase in enteric neurons results in postnatal intestinal dysmotility and dysbiosis. FASEB J..

[53] Takahashi T., Owyang C. (1995). Vagal control of nitric oxide and vasoactive intestinal polypeptide release in the regulation of gastric relaxation in rat. J. Physiol..

[54] Brack K.E., Patel V.H., Mantravardi R., Coote J.H., Ng G.A. (2009). Direct evidence of nitric oxide release from neuronal nitric oxide synthase activation in the left ventricle as a result of cervical vagus nerve stimulation. J. Physiol..

[55] Davis S.F., Derbenev A.V., Williams K.W., Glatzer N.R., Smith B.N. (2004). Excitatory and inhibitory local circuit input to the rat dorsal motor nucleus of the vagus originating from the nucleus tractus solitarius. Brain Res..

[56] Su Y., Xu J., Zhu Z., Chin J., Xu L., Yu H., Nudell V., Dash B., Moya E.A., Ye L. (2024). Brainstem Dbh(+) neurons control allergen-induced airway hyperreactivity. Nature.

[57] Valentino R.J., Chen S., Zhu Y., Aston-Jones G. (1996). Evidence for divergent projections to the brain noradrenergic system and the spinal parasympathetic system from Barrington’s nucleus. Brain Res..

[58] Bassi J.K., Connelly A.A., Butler A.G., Liu Y., Ghanbari A., Farmer D.G.S., Jenkins M.W., Melo M.R., McDougall S.J., Allen A.M. (2022). Analysis of the distribution of vagal afferent projections from different peripheral organs to the nucleus of the solitary tract in rats. J. Comp. Neurol..

[59] Tache Y., Bonaz B. (2007). Corticotropin-releasing factor receptors and stress-related alterations of gut motor function. J. Clin. Investig..

[60] Verstegen A.M.J., Vanderhorst V., Gray P.A., Zeidel M.L., Geerling J.C. (2017). Barrington’s nucleus: Neuroanatomic landscape of the mouse “pontine micturition center”. J. Comp. Neurol..

[61] Chen J., Cheng M., Wang L., Zhang L., Xu D., Cao P., Wang F., Herzog H., Song S., Zhan C. (2020). A Vagal-NTS Neural Pathway that Stimulates Feeding. Curr. Biol..

[62] Valentino R.J., Wood S.K., Wein A.J., Zderic S.A. (2011). The bladder-brain connection: Putative role of corticotropin-releasing factor. Nat. Rev. Urol..

